# Diverticulitis during pregnancy: A review of the reported cases

**DOI:** 10.3389/fmed.2022.942666

**Published:** 2022-11-11

**Authors:** Konstantinos S. Kechagias, Konstantinos Katsikas-Triantafyllidis, Georgios Geropoulos, Panagiotis Giannos, Marina Zafeiri, Imran Tariq-Mian, Maria Paraskevaidi, Anita Mitra, Maria Kyrgiou

**Affiliations:** ^1^Department of Metabolism, Digestion and Reproduction, Faculty of Medicine, Imperial College London, London, United Kingdom; ^2^Society of Meta-Research and Biomedical Innovation, London, United Kingdom; ^3^Department of General Surgery, NHS Foundation Trust, Royal Marsden Hospital, London, United Kingdom; ^4^Department of Life Sciences, Faculty of Natural Sciences, Imperial College London, London, United Kingdom; ^5^Imperial College Healthcare NHS Trust, London, United Kingdom

**Keywords:** pregnancy, diverticulitis, diverticular disease, acute abdomen, delivery

## Abstract

**Background:**

Diverticular disease of the colon represents a common clinical condition in the western world. Its prevalence increases with age and only 5% of cases occur in adults younger than 40 years of age, making it a rare condition during pregnancy. The aim of this review was to provide an overview of the reported cases of diverticulitis during pregnancy.

**Methods:**

We conducted a systematic review of the literature based on preferred reporting items for systematic review and meta-analysis (PRISMA) guidelines. We searched three different electronic databases namely PubMed, Scopus and Web of Science from inception to December 2021. Literature search and data extraction were completed in duplicates.

**Results:**

The initial search yielded 564 articles from which 12 were finally included in our review. Ten articles were case reports and two were observational studies. The mean age of the cases was 34 years. The presenting complain was provided for 11 cases. The majority of the patients (10/11, 91%) presented with abdominal pain located mainly on the left (6/11, 55%) or right (4/11, 36%) iliac fossa. The most common diagnostic modality used for the diagnosis of the condition was ultrasonography in nine cases (9/12, 75%) followed by magnetic resonance imaging (MRI) in two cases (2/12, 17%). In spite of clinical and radiological evaluation, the initial diagnosis was inaccurate in seven cases (7/12, 58%). The therapeutic approach was available for 11 cases and it was based on the administration of intravenous antibiotics in six cases (6/11, 55%) and surgical management in five cases (5/11, 45%). Data for the type of delivery was provided in nine studies with five patients (5/9, 56%) delivering vaginally and four patients (4/9, 44%) delivering with cesarean section.

**Conclusion:**

As advanced maternal age becomes more common, the frequency of diverticulitis in pregnancy may increase. Although available guidelines do not exist, the clinical awareness, early recognition of the disorder, using diagnostic modalities such as ultrasound and MRI, and rapid therapeutic approach with antibiotics, may improve maternal and neonatal outcomes.

## Introduction

Diverticulosis is defined as the anatomic change in the wall of gastrointestinal tract that is characterized by outpouching of the mucosa and submucosa through the muscularis (1). Diverticular disease, which develops at the base of diverticulosis, most commonly affects the colon, with the vast majority of cases located in the sigmoid (2). Its severity can range from asymptomatic, uncomplicated diverticular disease to symptomatic disease with complications such as acute inflammation (diverticulitis) or diverticular haemorrhage (3).

Although diverticular disease is common, its pathogenesis remains poorly understood (4). Epidemiological studies have shown an inverse relation between the incidence of diverticular disease and fiber content of the diet (5). Low dietary fiber decreases the volume of stool and prolongs transit time, leading to higher intraluminal pressures. Theoretically this high intraluminal pressure may cause herniation of colonic mucosa through areas of weakness (6).

Although recent reports suggest that the incidence of diverticulosis as well as diverticulitis has been increased especially in younger patients, the disease remains relatively rare before the age of 30 with its frequency increasing with advancing age (7, 8). Therefore, diverticulitis rarely affects gestation and is rarely considered as a differential diagnosis when managing pregnant women with abdominal symptoms (9). In addition, the decreased peritoneal signs that are encountered during gestation, further obscure the diagnosis of this entity in pregnant patients.

The aim of this systematic review was to provide a holistic overview of the currently available literature on the reported cases of acute diverticulitis during pregnancy and explore the diagnostic and therapeutic approaches used for the management of pregnant patients.

## Methods

This review was designed and conducted in accordance with the preferred reporting items for systematic review and meta-analysis (PRISMA) guidelines (10).

### Literature search and data extraction

PubMed, Scopus and Web of science were screened for articles published from inception till December 2021. After removal of duplicates, the remaining titles and abstracts were assessed for inclusion. Two authors (KK and KK-T) searched all databases independently and extracted data in pre-specified forms. Discrepancies in the literature search process and data extraction were discussed and resolved by GG.

There were no language and geographic region restrictions. The terms used for the PubMed search were: (diverticulitis OR diverticular disease OR diverticulosis) AND (pregnancy OR gestation). In Scopus and Web of science, the search was ensued using the aforementioned terms and was further limited based on study type to “articles.” Reference lists of relevant reviews and articles selected for inclusion were additionally manually searched. Two authors (KK and KK-T) extracted data independently on: name of first author, date of publication, country of origin, study design, number of subjects, age of patients, site of diverticulitis, presenting symptoms, diagnostic approach, treatment, gestational age and mode of delivery after the diagnosis of diverticulitis.

### Eligibility criteria

The systematic review included all studies which reported cases of diverticular disease or diverticulitis during pregnancy, irrespective of study design. *In vitro*, animal studies, conference abstracts and other non-peer reviewed sources were excluded from the review. Diagnosis of diverticulitis was established either clinically or using imaging modalities as described in the included studies.

### Quality assessment

The critical appraisal checklist for case reports provided by the Joanna Briggs Institute (JBI) was employed to evaluate the overall quality of the included studies (11). The assessment was performed based on the reporting of 8 different elements namely, patient demographics, medical history, health status, physical examination and diagnosis, concomitant therapies, post-intervention health status and drug administration reaction interface. The studies were scored either based on “Yes,” “No,” “Unclear or Not/Applicable” depending on the availability of information for every element.

## Results

### Study characteristics

**FIGURE 1:**
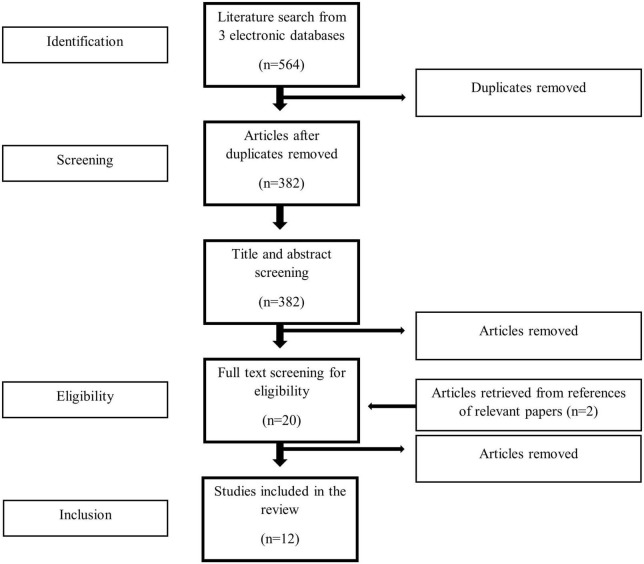
Preferred reporting items for systematic review and meta-analysis (PRISMA) workflow. Diagram of the selection process during the systematic review of the literature.

Of the 564 publications retrieved from the literature search, 10 studies were eligible for the systematic review. Two more studies were manually retrieved from references of relevant publications. In total, 12 articles were finally included (12–23). The selection process employed during the systematic literature search is described according to the PRISMA statement in Figure 1. In terms of design, ten studies were case reports and two were observational studies (one cohort and one cross-sectional). Four studies were conducted in North America, four in Europe, three in Asia and one in Africa. The characteristics of the included studies are shown in Table 1.

**TABLE 1:** Characteristics of the included studies.

References	Country	Study type	Age	Presenting symptoms	Gestational age (week)	Site	Differential diagnosis	First diagnostic tool	Second diagnostic tool	Accurate initial diagnosis	Treatment type	Mode of delivery (week)
Kasaven et al. (12)	UK	Case report	33	Pyrexia, LIF pain	24	Sigmoid colon	Pyelonephritis	Ultrasound	CT (post-delivery)	No	Antibiotics, loop ileostomy and pelvic washout (post-delivery)	Vaginal (25)
Kereshi et al. (13)	Israel	Cohort	N/A	RIF pain	N/A	Ileocecal	Appendicitis	MRI	No	No	Appendicectomy	N/A
Jung et al. (14)	Korea	Cross-sectional	N/A	N/A	N/A	N/A	Appendicitis	MRI	No	Yes	N/A	N/A
Milczarek-Łukowiak et al. (15)	Poland	Case Report	38	LIF pain	34	Sigmoid colon	Urolithiasis	Ultrasound	N/A	No	Exploratory laparotomy and abscess drainage combined with C-section	C-section (34)
Salah et al. (16)	Tunisia	Case Report	36	LIF pain, Pyrexia	28	Sigmoid colon	Peri-colic abscess complicating Crohn’s disease	Ultrasound	MRI	Yes	Antibiotics, sigmoidectomy with colorectal anastomosis (post-delivery)	Vaginal (32)
Shanbhogue et al. (17)	Canada	Case Report	30	RIF pain, nausea	11	Appendix	Appendicitis	Ultrasound	MRI	No	Appendicectomy	N/A
Bodner et al. (18)	Austria	Case Report	33	Right mid and lower abdominal pain, nausea and vomiting	37	Right colon	Appendicitis	Ultrasound	No	No	Antibiotics, Right hemicolectomy with ileo-transversostomy combined with C-section	C-section (37)
Ragu et al. (19)	France	Case Report	34	Fever, LIF and flank pain	29	Sigmoid colon	Pyelonephritis	Ultrasound	CT and MRI	Yes	Antibiotics	C-section (35)
Sherer et al. (20)	USA	Case Report	38	nausea, vomiting, LIF pain	33	Sigmoid colon	Degenerating leiomyoma	Ultrasound	MRI	Yes	Analgesics and hydration, Hartman’s (post-delivery)	Vaginal (34)
Pelosi et al. (21)	USA	Case Report	37	RUQ pain, nausea	20	Right colon	Haemorrhagic corpus luteum	Ultrasound	No	No	Antibiotics, Diagnostic laparoscopy	Vaginal (40)
Orihata et al. (22)	Japan	Case Report	28	RIF pain	15	Appendix	Appendicitis	Ultrasound	No	No	Appendicectomy	C-section (30)
Schnall et al. (23)	USA	Case Report	31	LIF pain	N/A	N/A	Ovarian cyst torsion, degenerating leiomyoma	IV pyelogram	Barium enema	Yes	Hydration	Vaginal (term)

N/A, not available; RIF, right iliac fossa; LIF, left iliac fossa; C-section, cesarean section; CT, Computer tomography; MRI, magnetic resonance imaging.

### Patient demographics

A total of 12 cases of diverticulitis during pregnancy were identified. The mean patient age was 34 years, based on available data from 10 studies. The mean gestational age at the time of diagnosis was 25 weeks, based on available data from nine studies.

### Clinical manifestation, diagnosis, and management

**FIGURE 2:**
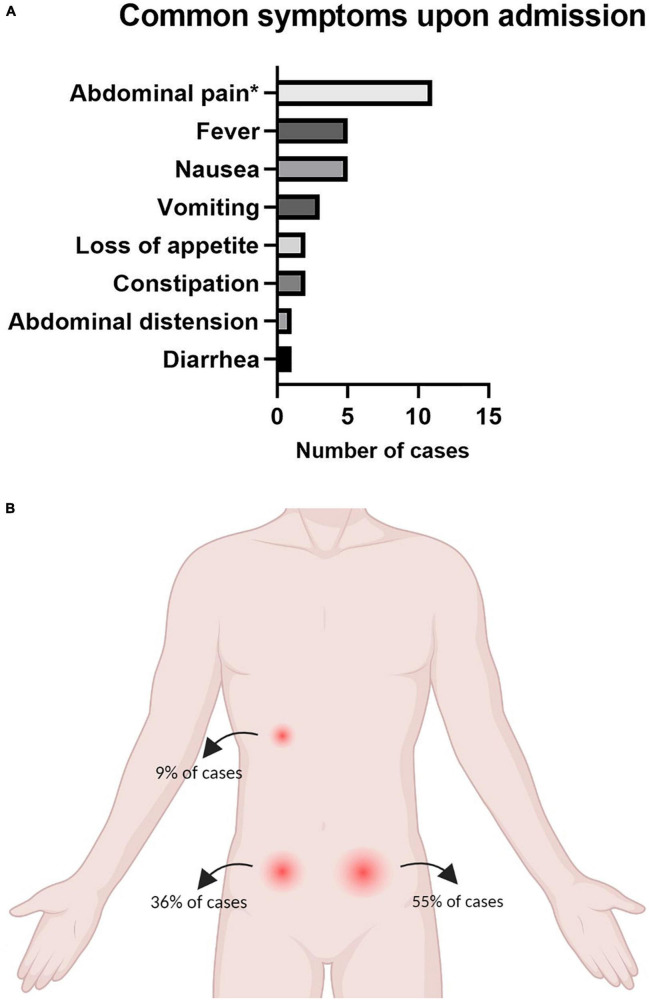
**(A)** Presenting symptoms of the 12 included cases of acute diverticulitis during pregnancy and **(B)** pain location at the time of presentation.

Data relevant to the presenting symptoms of diverticular disease was provided for 11 cases and is demonstrated in Figure 2A. The majority of patients presented with abdominal pain (10/11, 91%). Five (6/11, 55%) and four (4/11, 36%) patients presented with left and right iliac fossa pain, respectively, and one patient (1/11.9%) experienced right upper quadrant pain (Figure 2B). The second most common symptom of presentation was nausea and vomiting, encountered in five cases (5/11, 45%). Less common symptoms included abdominal distension and diarrhea.

Regarding the diagnostic approach employed, ultrasound was used as the first diagnostic modality in nine cases (9/12, 75%), and magnetic resonance imaging (MRI) as the initial imaging tool in two cases (2/12, 17%). In four cases (4/12, 33%) an inconclusive ultrasound was followed by an MRI. Despite the clinical and radiological evaluation, the initial diagnosis in seven cases was inaccurate (7/12, 58%), with five initially being diagnosed with acute appendicitis and two with pyelonephritis. In most of the patients, diverticular disease was located in the sigmoid colon (5/12, 42%) followed by the ascending colon (3/12, 25%) and appendix (2/12, 17%). Six women (6/12, 50%) were diagnosed during the third trimester, one (1/12, 8%) during the second trimester and one (1/12, 8%) during the first trimester. The gestational age at the time of diagnosis was not reported for the three remaining cases.

The therapeutic approach was available for 11 cases and it was based on the administration of intravenous antibiotics in six cases (6/11, 55%). Appendicectomy was performed in three cases (3/11, 27%) and simple bowel rest was used in one case (1/11, 9%). Laparotomy and drainage of intra-abdominal abscess with simultaneous cesarean section was performed in one case (1/11, 9%). Additionally, diverticulitis was treated surgically in three cases (3/11, 36%) after delivery due to failed medical management. The operations included right and left hemi-colectomy with anastomosis and Hartmann’s with loop ileostomy.

Data for the type of delivery was provided in nine studies with five patients (5/9, 56%) delivering vaginally and four patients (4/9, 44%) delivering with cesarean section. Two of them were performed as emergency cesarean sections at the time of the diagnostic laparotomy and two as elective procedures. Six of the deliveries (6/9, 67%) were preterm including three spontaneous preterm deliveries (3/9, 33%) and three preterm cesarean sections (3/9, 33%).

### Quality of the studies

Quality assessment of the eligible studies revealed that on average all of the recommended elements were fulfilled and thus, these were considered as low risk of bias. Only two studies did not attain a good score mainly due to inadequate availability of data (Table 2).

**TABLE 2:** Quality assessment of the included studies.

References	Q1	Q2	Q3	Q4	Q4	Q5	Q6	Q7	Q8
Kasaven et al. (12)	•	•	•	•	•	○	•	•	•
Jung et al. (14)	○	○	○	○	○	○	○	○	•
Kereshi et al. (13)	○	•	○	○	○	○	○	○	•
Milczarek-Łukowiak et al. (15)	•	•	•	•	•	•	•	•	•
Salah et al. (16)	•	•	•	•	•	•	•	•	•
Shanbhogue et al. (17)	•	•	•	•	•	•	○	•	•
Bodner et al. (18)	•	•	•	○	•	•	•	•	•
Ragu et al. (19)	•	○	•	•	•	•	○	•	•
Sherer et al. (20)	•	•	•	•	•	•	•	•	•
Pelosi et al. (21)	•	•	•	•	•	•	•	•	•
Orihata et al. (22)	•	•	•	•	•	•	•	•	•
Schnall et al. (23)	•	•	•	•	•	•	•	○	•

Q1: Were patient’s demographic characteristics clearly described?

Q2: Was the patient’s history clearly described and presented as a timeline?

Q3: Was the current clinical condition of the patient on presentation clearly described?

Q4: Were diagnostic tests or methods and the results clearly described?

Q5: Was the intervention(s) or treatment procedure(s) clearly described?

Q6: Was the post-intervention clinical condition clearly described?

Q7: Were adverse events (harms) or unanticipated events identified and described?

Q8: Does the case report provide takeaway lessons?

• = Yes; ○ = No.

## Discussion

Our study constitutes a contemporary systematic review of published cases of diverticular disease during pregnancy and provides an insight into the clinical manifestation and therapeutic management of this rare disorder during gestation. The current review was undertaken and reported using the PRISMA guidelines.

### Findings in context with the literature

The incidence of diverticulitis increases with age. Only 5% of cases affect patients younger than 40 years (24). However, the number of cases during pregnancy is expected to increase as more women delay their childbearing until later in life (25). A single centre analysis from the USA, which covered a 20-year period, reported an incidence around 1 in 6000 pregnancies (26). Diverticulitis is a well-established infectious cause for intra-abdominal sepsis, which may trigger cytokine release and consequently lead to preterm labour (27). Therefore, the disease should be always considered as part of the differential diagnosis of pregnant patients with abdominal symptoms.

Management of symptomatic diverticular disease during pregnancy should balance both fetal and maternal benefit. Nevertheless, due to the rarity of the disorder in the pregnant population, there are no established clinical protocols to achieve this balance. A recent review on the imaging modalities that can be used during pregnancy proposed that the investigation of pregnant women with abdominal symptoms should include the tools that are used in the diagnosis of appendicitis namely: ultrasonography and MRI (28). While ultrasonography plays a pivotal role in the imaging during pregnancy due to its safety profile, the expertise and experience of the radiologist who performs the scan should always be considered as a general limitation of the test (29, 30). In fact, in recent years there has been a shift toward non-contrast MRI for the evaluation of pregnant women with abdominal pain, either as a secondary test following an inconclusive ultrasound scan or as the primary test for some indications (31). Thus, an approach that initiates with a diagnostic ultrasound and continues with an MRI, seems to offer safety and relatively high sensitivity for intra-abdominal pathologies, including diverticulitis (32).

As far as the treatment is concerned, in the non-pregnant population, most cases of uncomplicated diverticulitis can be treated with antibiotics and dietary restrictions (6, 33). Amoxicillin and clavulanic acid constitute the first line of oral antibiotics followed by trimethoprim and metronidazole or cefalexin and metronidazole. Similarly, intravenous regimes include amoxicillin and clavulanic acid as first line followed by cefuroxime and metronidazole or amoxicillin and gentamicin and metronidazole. It is worth noting that the combination of amoxicillin and clavulanic acid should be avoided in third trimester due to the increased risk of necrotizing enterocolitis of the newborn (34). Similarly, trimethoprim should be avoided in first trimester due to folate antagonism (35). Finally, although quinolones are used in non-pregnant patients with diverticular disease are not routinely used during pregnancy and thus, consultation with a maternal–fetal medicine specialist should be considered (36).

Pregnancy should not be a contraindication to surgical management if this is warranted, for example, due to inadequate response to medical management. In a 2019 statement from the American College of Obstetrics and Gynaecology (ACOG) on non-obstetric surgery during pregnancy it was concluded that medically necessary surgery should not be denied in pregnancy due to the adverse effect that the disease itself may have on the pregnant woman and her fetus (37). Rates of fetal loss in pregnant women with acute appendicitis range from 1.5% with simple appendicitis (38) to 6% with generalized peritonitis and up to 36% with perforation (39), which is likely related to the infective and inflammatory insult from the disease itself. Whilst there is a lack of similar data relating to pregnancy outcomes in diverticulitis, it may well be similar, reinforcing the idea that surgical management in pregnancy should not be disregarded. In addition, ACOG concluded that there is a lack of evidence that *in utero* exposure to anaesthetic or sedative drugs has any negative impact on the fetal brain (37), which may be reassuring for both clinicians and patients.

Diet is another important aspect related to the management of patients with diverticular disease during pregnancy. During the period of gestation, the need for macro- and micro- nutrients as well as dietary fibre increases sharply to support a healthy pregnancy and childbirth (40). Adequate dietary fibre intake has been particularly linked with a reduced risk of gestational diabetes, preeclampsia, and constipation (41) and conceivably may prevent the reoccurrence of diverticulitis (5, 42). This has been attributed to the reduced contact time between bowel contents and diverticula which as a result reduces mucosal irritation (43, 44). The implementation of different dietary restrictions, from nil by mouth, to clear liquids has been also recommended in the past (45). The rationalization behind this is also associated with the reduction in mucosal irritation and inflammation secondary to decreased bowel motility. However, the aforementioned mechanism is not supported by published evidence and consequently, the dogma of a clear liquid diet has been abandoned by most recent guidelines (46).

### Strengths and limitations

The current review provides the only available overview of the clinical presentation, diagnosis, and therapeutic management of diverticular disease during pregnancy. Cases included in this review were identified from comprehensive search of databases using a systematic search approach and the quality of the included studies were assessed with scrutiny.

However, despite having applied stringent inclusion criteria, we were unable to rule out the possibility of missing some important cases aggregated in larger series. The small number of included studies constitutes a major limitation and also reinforces the view that diverticulitis during pregnancy is possibly an underreported condition in the literature. Thus, additional studies are required to reach safer conclusions about the optimal diagnostic and therapeutic management of the disease. Publication bias is another potential weak point as case reports of rare or atypical observations are more likely to be published, potentially excluding more common findings. A wider drawback involves the low-quality nature of case reports and series within our systematic review, which hampers the validity and interpretation of conclusions that can be attained. Therefore, their reported findings although appealing, may not reflect the truth without underlying valid description.

## Conclusion

Diverticulitis is a condition obstetricians may expect to see increase as advanced maternal age becomes more common. While available guidelines do not exist, the increased awareness of clinicians and the early recognition of the disorder, using diagnostic modalities, such as ultrasound and MRI, are crucial for the management of these patients. A rapid therapeutic approach with antibiotics may improve overall maternal and fetal outcomes, but surgical management should also be considered irrespective of gestation.

## Data availability statement

The original contributions presented in the study are included in the article/supplementary material, further inquiries can be directed to the corresponding author.

## Author contributions

KK and KK-T: conceptualization, investigation, resources, and writing—original draft preparation. KK, KK-T, and GG: methodology. KK, KK-T, GG, PG, MZ, IT-M, MP, AM, and MK: validation and writing—review and editing. KK: supervision. All authors have read and agreed to the published version of the manuscript.

## References

[1] Wan D Krisko T. Diverticulosis, diverticulitis, and diverticular bleeding. *Clin Geriatr Med.* (2021) 37:141–54. 10.1016/j.cger.2020.08.011 33213768

[2] Tursi A Scarpignato C Strate LL Lanas A Kruis W Lahat A Colonic diverticular disease. *Nat Rev Dis Primers.* (2020) 6:1–23. 10.1038/s41572-020-0153-5 32218442 PMC7486966

[3] Morris AM Regenbogen SE Hardiman KM Hendren S. Sigmoid diverticulitis: a systematic review. *JAMA.* (2014) 311:287–97. 10.1001/jama.2013.282025 24430321

[4] Janes SE Meagher A Frizelle FA. Management of diverticulitis. *Bmj.* (2006) 332:271–5. 10.1136/bmj.332.7536.271 16455722 PMC1360397

[5] Dahl C Crichton M Jenkins J Nucera R Mahoney S Marx W Evidence for dietary fibre modification in the recovery and prevention of reoccurrence of acute, uncomplicated diverticulitis: a systematic literature review. *Nutrients.* (2018) 10:137. 10.3390/nu10020137 29382074 PMC5852713

[6] Strate LL Morris AM. Epidemiology, pathophysiology, and treatment of diverticulitis. *Gastroenterology.* (2019) 156:1282–98.e1. 10.1053/j.gastro.2018.12.033 30660732 PMC6716971

[7] Miulescu MAM. Colonic diverticulosis. Is there a genetic component? *Mædica.* (2020) 15:105. 10.26574/maedica.2020.15.1.105 32419870 PMC7221275

[8] Bharucha AE Parthasarathy G Ditah I Fletcher J Ewelukwa O Pendlimari R Temporal trends in the incidence and natural history of diverticulitis: a population-based study. *Am J Gastroenterol.* (2015) 110:1589. 10.1038/ajg.2015.302 26416187 PMC4676761

[9] Masselli G Derme M Laghi F Framarino-dei-Malatesta M Gualdi G. Evaluating the acute abdomen in the pregnant patient. *Radiol Clin.* (2015) 53:1309–25. 10.1016/j.rcl.2015.06.013 26526440

[10] Liberati A Altman DG Tetzlaff J Mulrow C Gøtzsche PC Ioannidis JP The PRISMA statement for reporting systematic reviews and meta-analyses of studies that evaluate health care interventions: explanation and elaboration. *J Clin Epidemiol.* (2009) 62:e1–34. 10.1016/j.jclinepi.2009.06.006 19631507

[11] JB Institute. *The Joanna Briggs Institute Critical Appraisal Tools for use in JBI Systematic Review: Checklists for Case Reports.* Adelaide: The Joanna Briggs Institute (2019).

[12] Kasaven LS Karampitsakos T Todiwala A. Septiceamia and pre term labour due to severe diverticular abscess in pregnancy. *Eur J Obstet Gynecol Reprod Biol X.* (2019) 1:100006. 10.1016/j.eurox.2019.100006 31396593 PMC6683970

[13] Kereshi B Lee KS Siewert B Mortele KJ. Clinical utility of magnetic resonance imaging in the evaluation of pregnant females with suspected acute appendicitis. *Abdominal Radiol.* (2018) 43:1446–55. 10.1007/s00261-017-1300-7 28849364

[14] Jung JY Na JU Han SK Choi PC Lee JH Shin DH. Differential diagnoses of magnetic resonance imaging for suspected acute appendicitis in pregnant patients. *World J Emerg Med.* (2018) 9:26. 10.5847/wjem.j.1920-8642.2018.01.004 29290892 PMC5717372

[15] Milczarek-Łukowiak M Pyziak A Kocemba W Płusajska J. Complicated colonic diverticulitis at 34 weeks gestation. *Ginekol Pol.* (2012) 83:943–5. 23488299

[16] Salah RBH Mena KB Bourguiba MB Moussa MB Zaouche A. Sigmoïdite diverticulaire compliquée d’une fistule colo-tubaire survenant au cours d’une grossesse. *La Tunisie Med.* (2011) 89:574–5. 21681726

[17] Shanbhogue AKP Kielar A Nguyen B Shanbhogue DK Teo I. Appendiceal diverticulitis in pregnancy. *Eur J Radiol Extra.* (2009) 71:e29–31. 10.1016/j.ejrex.2009.01.001

[18] Bodner J Windisch J Bale R Wetscher G Mark W. Perforated right colonic diverticulitis complicating pregnancy at 37 weeks’ gestation. *Int J Color Dis.* (2005) 20:381–2. 10.1007/s00384-004-0643-z 15565304

[19] Ragu N Tichoux C Bouyabrine H Carabalona J Taourel P Bruel J Diverticulite sigmoïdienne compliquée en cours de grossesse. *J Radiol.* (2004) 85:1950–2. 10.1016/S0221-0363(04)97766-9 15602419

[20] Sherer DM Frager D Eliakim R. An unusual case of diverticulitis complicating pregnancy at 33 weeks’ gestation. *Am J Perinatol.* (2001) 18:107–12. 10.1055/s-2001-13639 11383700

[21] Pelosi M III Pelosi MA Villalona E. Right-sided colonic diverticulitis mimicking acute cholecystitis in pregnancy: case report and laparoscopic treatment. *Surg Laparosc Endoscopy.* (1999) 9:63–7. 10.1097/00019509-199901000-00015 9950133

[22] Orihata M Sasaki H Hata M Nakagawa H Kakegawa T Sagawa F. A case of appendiceal diverticulitis at 15 Weeks’ Gestation. *Jpn J Gastroenterol Surg.* (1998) 31:1893–6. 10.5833/jjgs.31.1893

[23] Schnall M Phaneuf L Conway J. Acute diverticulitis of the sigmoid in pregnancy. *Am J Obstet Gynecol.* (1945) 50:558–9. 10.1016/S0002-9378(16)40153-5

[24] Ferzoco LB Raptopoulos V Silen W. Acute diverticulitis. *N Engl J Med.* (1998) 338:1521–6. 10.1056/NEJM199805213382107 9593792

[25] Adachi T Endo M Ohashi K. Uninformed decision-making and regret about delaying childbearing decisions: a cross-sectional study. *Nurs Open.* (2020) 7:1489–96. 10.1002/nop2.523 32802369 PMC7424464

[26] Longo SA Moore RC Canzoneri BJ Robichaux A. Gastrointestinal conditions during pregnancy. *Clin Colon Rectal Surg.* (2010) 23:80–9. 10.1055/s-0030-1254294 21629625 PMC2967327

[27] Gilman-Sachs A Dambaeva S Salazar Garcia MD Hussein Y Kwak-Kim J Beaman K. Inflammation induced preterm labor and birth. *J Reprod Immunol.* (2018) 129:53–8. 30025845 10.1016/j.jri.2018.06.029

[28] Moreno CC Mittal PK Miller FH. Nonfetal imaging during pregnancy: acute abdomen/pelvis. *Radiol Clin North Am.* (2020) 58:363–80. 10.1016/j.rcl.2019.10.005 32044012

[29] Schreyer A Layer G. S2k guidlines for diverticular disease and diverticulitis: diagnosis, classification, and therapy for the radiologist. *RöFo.* (2015) 187:676–84. 10.1055/s-0034-1399526 26019048

[30] Cuomo R Barbara G Pace F Annese V Bassotti G Binda GA Italian consensus conference for colonic diverticulosis and diverticular disease. *United Eur Gastroenterol J.* (2014) 2:413–42. 10.1177/2050640614547068 25360320 PMC4212498

[31] Shur J Bottomley C Walton K Patel JH. Imaging of acute abdominal pain in the third trimester of pregnancy. *Bmj.* (2018) 361:k2511. 10.1136/bmj.k2511 29929950

[32] Liljegren G Chabok A Wickbom M Smedh K Nilsson K. Acute colonic diverticulitis: a systematic review of diagnostic accuracy. *Colorectal Dis.* (2007) 9:480–8. 10.1111/j.1463-1318.2007.01238.x 17573739

[33] NICE Guideline. *Diverticular Disease: Diagnosis and Management.* London: National Institute for Health and Care Excellence (NICE) (2019). 32525625

[34] Nahum GG Uhl K Kennedy DL. Antibiotic use in pregnancy and lactation: what is and is not known about teratogenic and toxic risks. *Obstet Gynecol.* (2006) 107:1120–38. 10.1097/01.AOG.0000216197.26783.b5 16648419

[35] Gleckman R Blagg N Joubert DW. Trimethoprim: mechanisms of action, antimicrobial activity, bacterial resistance, pharmacokinetics, adverse reactions, and therapeutic indications. *Pharmacotherapy.* (1981) 1:14–9. 10.1002/j.1875-9114.1981.tb03548.x 6985448

[36] Yefet E Schwartz N Chazan B Salim R Romano S Nachum Z. The safety of quinolones and fluoroquinolones in pregnancy: a meta-analysis. *Bjog.* (2018) 125:1069–76. 29319210 10.1111/1471-0528.15119

[37] Tolcher MC Fisher WE Clark SL. Nonobstetric surgery during pregnancy. *Obstet Gynecol.* (2018) 132:395–403. 10.1097/AOG.0000000000002748 29995718

[38] Babaknia A Parsa H Woodruff JD. Appendicitis during pregnancy. *Obstet Gynecol.* (1977) 50:40–1. 10.1055/s-0040-1708849 876520

[39] Silvestri MT Pettker CM Brousseau EC Dick MA Ciarleglio MM Erekson EA. Morbidity of appendectomy and cholecystectomy in pregnant and non-pregnant women. *Obstet Gynecol.* (2011) 118:1261. 10.1097/AOG.0b013e318234d7bc 22105255 PMC3702040

[40] Potdar RD Sahariah SA Gandhi M Kehoe SH Brown N Sane H Improving women’s diet quality preconceptionally and during gestation: effects on birth weight and prevalence of low birth weight–a randomized controlled efficacy trial in India (Mumbai Maternal Nutrition Project). *Am J Clin Nutr.* (2014) 100:1257–68. 10.3945/ajcn.114.084921 25332324 PMC4196482

[41] Zerfu TA Mekuria A. Pregnant women have inadequate fiber intake while consuming fiber-rich diets in low-income rural setting: evidences from Analysis of common “ready-to-eat” stable foods. *Food Sci Nutr.* (2019) 7:3286–92. 10.1002/fsn3.1188 31660142 PMC6804770

[42] Strate LL Keeley BR Cao Y Wu K Giovannucci EL Chan AT. Western dietary pattern increases, and prudent dietary pattern decreases, risk of incident diverticulitis in a prospective cohort study. *Gastroenterology.* (2017) 152:1023–30.e2. 10.1053/j.gastro.2016.12.038 28065788 PMC5367955

[43] Carabotti M Annibale B Severi C Lahner E. Role of fiber in symptomatic uncomplicated diverticular disease: a systematic review. *Nutrients.* (2017) 9:161. 10.3390/nu9020161 PMC5331592 28230737

[44] Slavin J. Fiber and prebiotics: mechanisms and health benefits. *Nutrients.* (2013) 5:1417–35. 10.3390/nu5041417 23609775 PMC3705355

[45] Quigley EM Fried M Gwee KA Khalif I Hungin AP Lindberg G World gastroenterology organisation global guidelines irritable bowel syndrome: a global perspective update September 2015. *J Clin Gastroenterol.* (2016) 50:704–13. 10.1097/MCG.0000000000000653 27623513

[46] Schultz J Azhar N Binda G Barbara G Biondo S Boermeester M European Society of Coloproctology: guidelines for the management of diverticular disease of the colon. *Colorectal Dis.* (2020) 22:5–28. 10.1111/codi.15140 32638537

